# Emerging roles of cardiolipin remodeling in mitochondrial dysfunction associated with diabetes, obesity, and cardiovascular diseases

**DOI:** 10.1016/S1674-8301(10)60003-6

**Published:** 2010-01

**Authors:** Yuguang Shi

**Affiliations:** Department of Cellular and Molecular Physiology, Pennsylvania State University College of Medicine, Hershey, PA 17033, U.S.A.

## Abstract

Cardiolipin (CL) is a phospholipid exclusively localized in inner mitochondrial membrane where it is required for oxidative phosphorylation, ATP synthesis, and mitochondrial bioenergetics. The biological functions of CL are thought to depend on its acyl chain composition which is dominated by linoleic acids in metabolically active tissues. This unique feature is not derived from the *de novo* biosynthesis of CL, rather from a remodeling process that involves in phospholipases and transacylase/acyltransferase. The remodeling process is also believed to be responsible for generation of CL species that causes oxidative stress and mitochondrial dysfunction. CL is highly sensitive to oxidative damages by reactive oxygen species (ROS) due to its high content in polyunsaturated fatty acids and location near the site of ROS production. Consequently, pathological remodeling of CL has been implicated in the etiology of mitochondrial dysfunction commonly associated with diabetes, obesity, heart failure, neurodegeneration, and aging that are characterized by oxidative stress, CL deficiency, and abnormal CL species. This review summarizes recent progresses in molecular, enzymatic, lipidomic, and metabolic studies that support a critical regulatory role of pathological CL remodeling as a missing link between oxidative stress and mitochondrial dysfunction in metabolic diseases and aging.

## INTRODUCTION

Mitochondrial dysfunction has recently been identified as a common metabolic defect associated with obesity and its metabolic complications[1],[2]. A number of early studies suggest that mitochondrial oxidative function was compromised in diabetic and prediabetic humans as evidenced by reduced levels of fatty acid oxidation[3], insulin-stimulated ATP synthesis[4]–[6], and expression of genes involved in oxidative phosphorylation[7]. However, this hypothesis has recently been challenged by findings that mitochondrial hyperactivity is associated with severe insulin resistance in Asian Indians[8]. Attenuation of oxidative phosphorylation activity prevents the onset of diet-induced obesity and its related insulin resistance in mice with targeted deletion of AIF, a mitochondrial flavoprotein apoptosis inducing factor[9]. Furthermore, all of the insulin sensitizers, including some of the most popular antidiabetic drugs thiazolidinediones and metformin, have been shown to suppress mitochondrial complex I activity[10]–[13]. Therefore, the molecular mechanisms underlying a causative role of mitochondrial dysfunction in diabetes and obesity remain to be elucidated.

Cardiolipin(CL) is polyglycerophospholipid exclusively localized in the mitochondria where it regulates mitochondrial function and oxidative stress in species from yeast to mammals[14]–[16]. This role is mediated by the acyl composition of the side chains of CL, which is dominated by linoleic acid in insulin sensitive tissues[17]. This unique acyl composition is not derived from *de novo* synthesis of CL, rather from a remodeling process that involves phospholipases and acyltransferase/transacylases[18]–[20]. This remodeling process is also believed to be responsible for generating CL species that are highly sensitive to oxidative damage by reactive oxygen species (ROS), further exacerbating CL peroxidation and oxidative stress. CL is sensitive to damage of its double bonds by oxidative stress due to its rich content in linoleic acid and its location near the site of ROS production in the inner mitochondrial membrane. CL is the only phospholipid in mitochondria that undergoes early oxidation during apoptosis[21]. Consequently, pathological CL remodeling has been implicated in etiology of mitochondrial dysfunction associated with a host of pathophysiological conditions including diabetes, obesity, heart failure, hyperthyroidism, neurodegeneration, and aging, all of which are characterized by increased levels of oxidative stress, CL deficiency, and enrichment of docosahexaenoic acid (DHA) content in CL[22]–[26]. Recent progress in molecular cloning of enzymes involved in CL synthesis and remodeling, combined with the latest development in lipidomic profiling of CL, have implicated an important role of CL remodeling in regulating health and diseases.

## CL SYNTHESIS, REMODELING, AND MITOCHONDRIAL FUNCTION

**Fig. 1:**
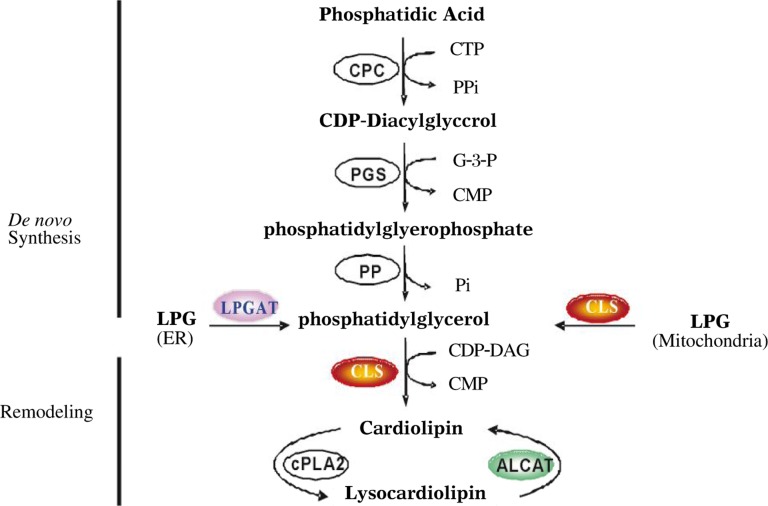
CL biosynthetic and remodeling pathways. The indicated reactions of CL biosynthetic pathway are catalyzed by the following enzymes: CTP-phosphatidic acid cytidylytransferase (CPC), phosphatidylglycerolphosphate synthase (PGS), phosphatidylglycerolphosphate phosphatase (PP), and CL synthase (CLS). In addition to CL synthesis, CLS is also involved in phosphatidylglycerol remodeling by catalyzing acylation of lysophosphatidylglycerol (LPG) to phosphatidylglycerol. Phosphatidylglycerol remodeling can also by catalyzed by LPG acyltransferase (LPGAT1). The CL remodeling pathway is catalyzed by phospholipase (cPLA) and acyl-CoA:lysoCL acyltransferase (ALCAT).

CL is a mitochondrial membrane phospholipid initially identified from beef heart, and is required for optimal mitochondrial respiration as a cofactor of enzymes involved in oxidative phosphorylation. CL is synthesized by three consecutive steps beginning with the biosynthesis of CDP-diacylglycerol. The committed and rate-limiting step is catalyzed by phosphatidylglycerophosphate synthase (PGS) (***Fig. 1***). CL is required for the reconstituted activity of a number of metabolic enzymes and carrier proteins in the mitochondria[17]. CL in the inner mitochondrial membrane serves as a Ca^2+^-binding site, through which Ca^2+^ triggers mitochondrial membrane permeabilization[27]. Additionally, CL is required for cell survival, and dissociation of cytochrome c from CL triggers apoptosis[28],[29]. In the yeast *S. cerevisiae*, mutation of the *crd1* gene encoding CL synthase results in impaired viability, a decrease in membrane potential, and defective oxidative phosphorylation[30]. Similarly, CL deficiency in Chinese hamster ovary (CHO) cells results in stringent temperature sensitivity for cell growth in glucose-deficient medium and by reduced ATP production[31]. The mutant CHO cells demonstrate an increased glycolysis, reduced oxygen consumption, and defective respiratory electron transport chain activity.

CL is the only known dimeric phospholipid, consisting of four fatty acyl chains, which is restricted to C18 chains dominated by the linoloeyl group (C18:2) in skeletal muscle and heart[32]. The unique fatty acyl composition is believed to be important for its proper biological function. The hydrophobic double-unsaturated linoleic diacylglycerol species is required for high affinity binding of CL to proteins[33]. Thus, alteration in molecular species composition of CL affects the activities of cytochrome *c* oxidase and other electron transport chain enzymes[29],[34]. However, the formation of the unique fatty acyl composition of CL does not occur during *de novo* biosynthesis, because the enzymes of the CL biosynthetic pathway lack appropriate substrate selectivity[35]–[37]. This is further confirmed by the recent cloning of the human CL synthase gene from our lab and others[38]–[40]. Thus, newly synthesized CL is believed to undergo a remodeling process to achieve its appropriate acyl content. In addition to CL synthesis, we have recently demonstrated that the human CLS1 is also involved in the remodeling of phosphatidylglycerol[41]. The recombinant hCLS1 protein expressed in COS-7 cells and Sf-9 insect cells exhibited a strong acyl-CoA dependent lysophosphatidylglycerol acyltransferase activity[41].

**Fig. 2:**
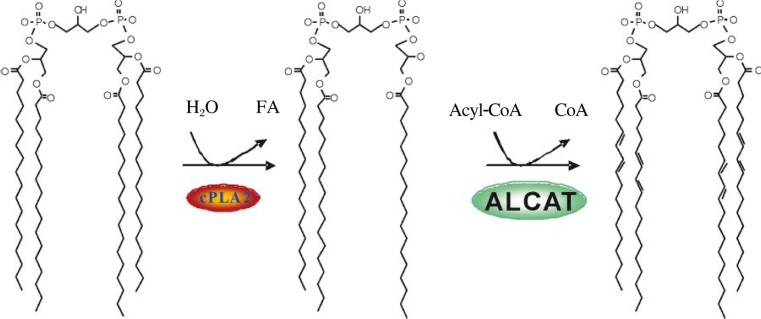
Proposed remodeling pathway for CL. CL is first deacylated to monolysoCL by phospholipase A2 (PLA2), and then reacylated to CL with ALCAT.

Two distinct mechanisms have been posited to carry out the CL remodeling process. The first mechanism involves transacylation of acyl groups from phosphatidylcholine or phosphatidylethanolamine to CL, which is partly catalyzed by tafazzin, an enzyme that when mutated causes defective CL remodeling and Barth syndrome[18] (see below). The alternative pathway involves deacylation by phospholipase A2 to lysoCL followed by reacylation to CL by acyl-CoA dependent Lyso-CL acyltransferases (***Fig. 2***)[17]. Two acyltransferases has been characterized so far, and they differ in substrate specificity and intracellular localization. In the search for a gene encoding an acyltransferase responsible for CL remodeling, we have recently identified and characterized the first CL reacylation enzyme, named acyl-CoA:lysoCL acyltransferase (ALCAT1)[19]. The recombinant ALCAT1 enzyme is localized in endoplasmic reticulum and recognizes both MLCL and dilysoCL as substrates. The second acyltransferase was a monolysoCL acyltransferase initially purified from pig liver mitochondria, known as MLCL AT, that catalyzes the synthesis of tetralinoleoyl-CL (L4CL)[42]. The gene encoding MLCL AT has recently been identified to share the same sequence homology with the mitochondrial trifunctional protein[20]. In comparison to the MLCL AT, ALCAT1 lacks preference for linoleic acid as substrate, suggesting a possible role of ALCAT1 in pathological remodeling of CL[19],[20]. This is corroborated by our recent reports that ALCAT1 expression is up-regulated in mammalian cells exhibiting tetralinoleoyl-CL deficiency and in heart and liver of mice suffering from oxidative stress induced by hyperthyroidism[43]. However, it remains to be identified whether ALCAT1 plays a causative role in pathological remodeling of CL.

## OXIDATIVE STRESS AND CL PEROXIDATION

**Fig. 3:**
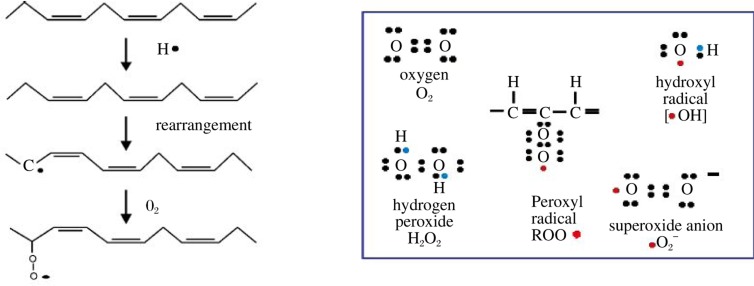
ROS and lipid peroxidation. ROS contain free radicals from atoms that have one or more unpaired electron(s) (right panel). The imbalance in electrons results in the high reactivity of the free radicals. The process of lipid peroxidation (left panel) begins with the ROS attack on double bounds of PUFA. The carbon radical tends to be stabilized by a molecular rearrangement to form a conjugated diene. Under aerobic conditions conjugated dienes are able to combine with O_2_ to produce a peroxyl radical, ROO^−^.

One of the common defects associated with metabolic diseases (diabetes, obesity, cardiovascular diseases), aging, and neurodegeneration is an increased level of oxidative stress. Increased level of ROS production has been implicated in the onset of mitochondrial dysfunction and is believed to the primary causes of diabetic complications[44]. CL is particularly sensitive to oxidation of its double bonds by ROS due to its location near the site of ROS production in the inner mitochondria membrane[28]. The mitochondrial electron transport chain is considered a major intracellular source of ROS including hydroxyl radicals, peroxy radicals, superoxides, and the dismutation product, H_2_O_2_ (***Fig. 3***). All of these ROS are generated both during physiologic respiration and during disrupted electron transport[28],[29]. Oxygen free radicals are highly reactive species capable of causing oxidation of CL, a process also known as lipid peroxidation. Non-oxidized CL is required for the mitochondrial bioenergetics and the activity of key mitochondrial proteins[28]. Consequently, CL peroxidation by ROS disrupts its binding with cytochrome *c* and affects the activity of complex I, III, and IV of the mitochondrial respiratory chain[45]. A burst of ROS damages mitochondria by causing profound loss of CL[46]. CL deficiency in ischemia and reperfusion results in mitochondrial dysfunction manifested by a decreased oxidative capacity, loss of cytochrome *c*, and generation of ROS. CL, but not its peroxidized form, is able to almost completely restore the ROS-induced loss of cytochrome *c* oxidase activity[47]. In support of a key role of CL peroxidation in mitochondrial dysfunction, CL is the only phospholipid that undergoes oxidation during the onset of apoptosis[21].

## PATHOLOGICAL CL REMODELING AS A COMMON DEFECT IN MITOCHONDRIAL DYSFUNCTION IN METABOLIC AND AGING-RELATED DISEASES

### Bath Syndrome(BTHS)

One of the best examples that underscores the importance of CL remodeling in metabolic diseases is BTHS, an X-linked recessive disorder manifested by cardiomyopathy, skeletal myopathy, growth retardation, and neutropenia[48]. BTHS is caused by mutations in the *tafazzin* (*TAZ*) gene encoding an acyltransferase involved in the remodeling of phospholipids[49]. The lipid composition of cells from patients with BTHS shows a dramatic decrease in CL levels and reduced incorporation of linoleic acid (18:2) into CL and its precursor phosphatidylglycerol, even though their biosynthetic capacity to synthesize CL remains unchanged[50],[51]. In addition, tetralinoleoyl-CL, the most predominant CL species in mitochondria from normal skeletal and heart muscle, is almost completely absent in BTHS, whereas the content and the acyl composition of other phospholipids are not affected[52]. Mitochondria of BTHS patients exhibit abnormal ultrastructure and respiratory chain defects in muscle and fibroblasts[48],[53].

### Diabetes and obesity

Diabetes and obesity are characterized by systemic oxidative stress which is believed to be a principal causative factor of insulin resistance and other obesity-related metabolic complications[44],[54]–[57]. ROS production was significantly increased in obesity and diabetes from elevated expression of NADPH oxidase and decreased expression of antioxidative enzymes[55],[56],[58]. The level of superoxide dismutase, the enzyme responsible for inactivating the superoxide radical, along with the levels of antioxidants are decreased in uncontrolled diabetes[59]. Oxidative stress impaired glucose uptake in muscle and fat[60],[61], and was recently shown to be the primary cause of various forms of insulin resistance[57]. Consistent with the notion of mitochondria as the primary source of ROS, employment of mitochondrial-targeted antioxidants ameliorated insulin resistance associated with obesity in both rodents and humans[54],[57],[62]. Many of the mitochondrial defects associated with diabetes were reversed by transgenic overexpression of catalase in mice[62].

Pathological CL remodeling contributes to the onset of mitochondrial dysfunction and metabolic complications associated with obesity. Diabetes and obesity are associated with CL depletion in myocardium and linked with altered substrate utilization and mitochondrial dysfunction[22]. Defective CL remodeling in the diabetic heart results in enrichment of DHA (22:6n3) in CL[63], which is known to cause mitochondrial dysfunction. Accumulation of DHA and other long chain polyunsaturated fatty acid(PUFA) in CL increases oxidant production and mitochondrial proton leakage in cultured mammalian cells[64],[65]. Treatment of diabetic mice with rosiglitazone, an insulin sensitizing drug, significantly increases CL levels and causes a substantial remodeling of CL toward an elevated linoleic acid (18:2n6) and a reduction of DHA content[63],[66]. Such a shift is believed to improve electron transport efficiency and decrease proton leakage[31],[65]. For example, when acyl composition of rat heart CL switched from 18:2n6 to 22:6n3, the activity of cytochrome *c* oxidase decreased by 50%, concurrent with a lower oxygen consumption rate of rat heart mitochondria[67].

### Hyperthyroidism

Thyroid hormone is a major physiological modulator of oxidative stress and mitochondrial respiration[68]. Thyroid hormone has been shown to increase mitochondrial mass, mitochondrial cytochrome *c* content, respiratory rate, and capacity of oxidative metabolism[69]. Hyperthyroidism is associated with significant mitochondrial dysfunction. Cardiovascular tissues are particularly sensitive to ROS damage associated with hyperthyroidism, because of the high energy demand of the heart. For example, hyperthyroid hearts displayed tachycardia and low functional recovery. Their mitochondria exhibited higher level of H_2_O_2_ production and susceptibility to swelling during reperfusion[70]. Both the levels of CL and lipid composition are profoundly altered by thyroid hormone status. Hyperthyroidism and hypothyroidism reciprocally affect the level of oxidative stress, lipid peroxidation, CL synthesis and remodeling[71],[72]. The hepatic and cardiac CL contents were elevated in rats treated by thyroxine, which was accompanied by an increase in CL synthase activity and level of ROS production[73],[74]. Moreover, cardiac mitochondrial MLCL AT activity was stimulated in hyperthyroid rats[75], and decreased in rats made hypothyroid[76]. Consistent with increased level of ROS production, hyperthyroidism is associated with a marked loss of C18:2, concurrent with a significant increases in polyunsaturated fatty acids such as arachidonic acid (C20:4) and DHA (C22:6)[72]. These changes increased the double bond index by 27% and CL peroxidizibility by 266%, which is likely to contribute to the elevated level of oxidative stress associated with hyperthyroidism[77]. In support of a possible role of ALCAT1 in pathological remodeling of CL, ALCAT1 mRNA expression was significantly up-regulated by hyperthyroidism and down-regulated by hypothyroidism[78].

### Heart Failure

Mitochondria in the adult mammalian heart have a tremendous capacity for oxidative metabolism, and the conversion of energy by these pathways is critical for proper cardiac function. Mitochondrial ROS in the heart has been reported to increase with age[79]. A number of endogenous mitochondrial antioxidant defenses may also diminish with age and thus reduce the capacity for efficient management of ROS[80]. CL is one of the principle phospholipids in the mammalian heart, a tissue that has perpetually high energy demands and is particularly sensitive to oxidative stress and mitochondrial dysfunction. Consequently, CL deficiency in ischemia and reperfusion results in mitochondrial dysfunction manifested by a decrease in oxidative capacity, loss of cytochrome *c*, and generation of ROS[47],[81],[82]. CL, but not the peroxidized form, was able to almost completely restore the ROS-induced loss of cytochrome c oxidase activity[47]. CL is also an immunogenic phospholipid, and development of anti-CL antibodies is associated with the onset of thrombosis[83]. Loss of cardiac tetralinoleoyl CL has recently been identified to be a major defect in experimental heart failure[84].

### Aging

Oxidative injury of mitochondria impacts critical aspects of the aging process and contributes to impaired physiological function, and has been proposed as the primary cause of aging. Mitochondrial dysfunction plays a central role in a wide range of aging-related disorders and various forms of cancer, resulting in a reduced life span[85],[86]. In support of a causative role of oxidative stress in aging, ROS levels and phospholipid peroxidation index are inversely correlated with life span, from mice to human[87]–[89]. Oxidative stress is also believed to contribute to an aging-associated decline in CL. Consistent with an increased level of ROS production, aging is associated with CL deficiency and profound remodeling of CL similar to that observed in metabolic diseases. Aging and physical exercise reciprocally affect mitochondrial and cardiac function by regulating CL levels in the heart. Exercise increases insulin sensitivity and the level of tetralinoleoyl CL, whereas aging decreases tetralinoleoyl CL level with concurrent increase in the DHA content in CL[25],[26],[84]. Aging has been shown to decrease CL content in heart, liver, and kidney. Aging related loss of CL impairs mitochondrial function by decreasing the activity of mitochondrial phosphate transporter, pyruvate carrier, adenine nucleotide transporter, and cytochrome oxidase, all of which requires CL for optimum activity[90]–[94]. These defects can be restored by supplementation of acyl-carnitine which is believed to restore CL levels[16].

### Neurological Diseases

Oxidative damage of mitochondrial function is implicated in the neurodegenerative process, and contributes to the onset of Parkinson's and Alzheimer's diseases[95]. The rates of neurodegeneration are strongly correlated with rates of formation of mitochondrial reactive oxygen and nitrogen species[89]. CL levels in the brain decrease with aging, which is likely to be caused by peroxidation from oxidative stress[96],[97]. In contrast to metabolically active tissues, such as skeletal muscle and heart, the tetralinoleoyl CL is not the predominant form of CL in the brain, representing less than 5% of the total CL[98]. Hence, CL from mouse brain is dominated by long chain PUFA, including DHA (C22:6) and arachidonic acids (C20:4) which contribute to 40% of the acyl side chains of CL[98],[99]. Although the biological significance of the acyl composition remains elusive, the high content in PUFA renders CL in the brain highly sensitive to oxidative damage. Consequently, traumatic brain injury has been shown to selectively increase the content of DHA in CL and cause CL peroxidation[100]. This selective CL peroxidation preceded peroxidation of other phospholipids and the onset of apoptosis[100]. Furthermore, mice with targeted inactivation of presynaptic protein, α-synuclein, a protein implicated in Parkinson's disease, exhibited CL deficiency and a reduction in both tetralinoleoyl CL content and mitochondrial complex I/III activity of the electron transport chain[99]. The knockout mice also exhibited deficiency of PG, the precursor for CL synthesis.

## CONCLUSION

**Fig. 4:**
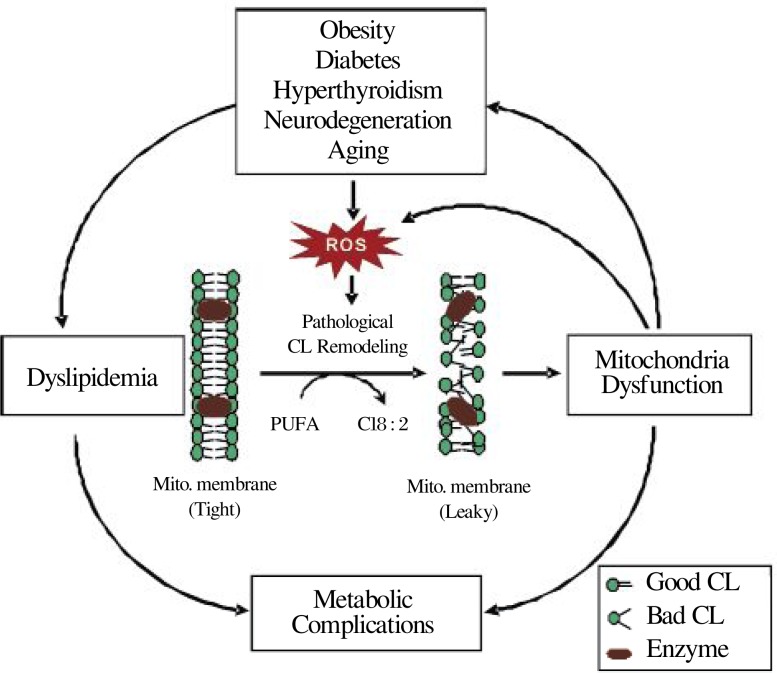
Pathological remodeling of CL as a common denominator of mitochondrial dysfunction in metabolic and aging-related diseases. Metabolic, neurodegenerative, and aging-related disorders increase the level of ROS production which causes CL peroxidation and pathological remodeling of CL. Pathological remodeling of CL replaces linoleic acid (C18:2) with PUFA, leading to mitochondrial proton leakage, oxidative stress, and mitochondrial dysfunction which further exacerbates ROS production, metabolic complications, and the pathophysiology of the diseases.

There is now a growing body of evidence that supports a causative role of pathological remodeling of CL in mitochondrial dysfunction in metabolic and neurological disorders and aging. Collectively, the information presented in this review has implicated an important role of pathological CL remodeling as a missing link between oxidative stress and mitochondrial dysfunction associated with various pathological conditions, including diabetes, obesity, hyperthyroidism, heart failure, BTHS, neurological diseases, and aging (***Fig. 4***). Although much of the evidence accumulated thus far remains circumstantial and descriptive, the latest development in highly sensitive analytical methods for CL profiling combined with major progress in the identification and cloning of genes encoding CL remodeling enzymes signals a new dawn of this exciting field of research. It can be envisaged that pathological remodeling of CL might be the common denominator of mitochondrial dysfunction of all the aging-related diseases. Consequently, targeting enzymes involved in pathological remodeling of CL by chemical compounds could provide novel treatments for metabolic diseases and aging.

## References

[1] Lowell BB, Shulman GI (2005). Mitochondrial dysfunction and type 2 diabetes. Science.

[2] Morino K, Petersen K F, Shulman GI (2006). Molecular mechanisms of insulin resistance in humans and their potential links with mitochondrial dysfunction. Diabetes.

[3] Bandyopadhyay GK, Yu JG, Ofrecio J, Olefsky JM (2006). Increased malonyl-CoA levels in muscle from obese and type 2 diabetic subjects lead to decreased fatty acid oxidation and increased lipogenesis; thiazolidinedione treatment reverses these defects. Diabetes.

[4] Asmann YW, Stump CS, Short KR, Coenen-Schimke JM, Guo Z, Bigelow ML (2006). Skeletal muscle mitochondrial functions, mitochondrial DNA copy numbers, and gene transcript profiles in type 2 diabetic and nondiabetic subjects at equal levels of low or high insulin and euglycemia. Diabetes.

[5] Petersen KF, Dufour S, Shulman GI (2005). Decreased insulin-stimulated ATP synthesis and phosphate transport in muscle of insulin-resistant offspring of type 2 diabetic parents. PLoS Med.

[6] Stump CS, Short KR, Bigelow ML, Schimke JM, Nair KS (2003). Effect of insulin on human skeletal muscle mitochondrial ATP production, protein synthesis, and mRNA transcripts. Proc Natl Acad Sci USA.

[7] Mootha VK, Handschin C, Arlow D, Xie X, St Pierre J, Sihag S (2004). Erralpha and Gabpa/b specify PGC-1alpha-dependent oxidative phosphorylation gene expression that is altered in diabetic muscle. Proc Natl Acad Sci USA.

[8] Nair KS, Bigelow ML, Asmann YW, Chow LS, Coenen-Schimke JM, Klaus KA (2008). Asian Indians have enhanced skeletal muscle mitochondrial capacity to produce ATP in association with severe insulin resistance. Diabetes.

[9] Pospisilik JA, Knauf C, Joza N, Benit P, Orthofer M, Cani PD (2007). Targeted deletion of AIF decreases mitochondrial oxidative phosphorylation and protects from obesity and diabetes. Cell.

[10] Turner N, Li JY, Gosby A, To SWC, Cheng Z, Miyoshi H (2008). Berberine and its more biologically available derivative, dihydroberberine, inhibit mitochondrial respiratory complex I: a mechanism for the action of berberine to activate AMP-activated protein kinase and improve insulin action. Diabetes.

[11] Brunmair B, Staniek K, Gras F, Scharf N, Althaym A, Clara R (2004). Thiazolidinediones, like metformin, inhibit respiratory complex I: a common mechanism contributing to their antidiabetic actions?. Diabetes.

[12] Owen MR, Doran E, Halestrap AP (2000). Evidence that metformin exerts its anti-diabetic effects through inhibition of complex 1 of the mitochondrial respiratory chain. Biochem J.

[13] El-Mir MY, Nogueira V, Fontaine E, Averet N, Rigoulet M, Leverve X (2000). Dimethylbiguanide inhibits cell respiration via an indirect effect targeted on the respiratory chain complex I. J Biol Chem.

[14] Chen S, He Q, Greenberg ML (2008). Loss of tafazzin in yeast leads to increased oxidative stress during respiratory growth. Mol Microbiol.

[15] Chicco AJ, Sparagna GC (2007). Role of cardiolipin alterations in mitochondrial dysfunction and disease. Am J Physiol Cell Physiol.

[16] Paradies G, Petrosillo G, Paradies V, Ruggiero FM (2009). Role of cardiolipin peroxidation and Ca2+ in mitochondrial dysfunction and disease. Cell Calcium.

[17] Schlame M, Rua D, Greenberg ML (2000). The biosynthesis and functional role of cardiolipin. Prog Lipid Res.

[18] Xu Y, Kelley RI, Blanck TJJ, Schlame M (2003). Remodeling of cardiolipin by phospholipid transacylation. J Biol Chem.

[19] Cao J, Liu Y, Lockwood J, Burn P, Shi Y (2004). A novel cardiolipin-remodeling pathway revealed by a gene encoding an endoplasmic reticulum-associated acyl-CoA:lysoCL acyltransferase (ALCAT1) in mouse. J Biol Chem.

[20] Taylor WA, Hatch GM (2009). Identification of the human mitochondrial linoleoyl-coenzyme A monolysocardiolipin acyltransferase (MLCL AT-1). J Biol Chem.

[21] Kagan VE, Tyurin VA, Jiang J, Tyurina YY, Ritov VB, Amoscato AA (2005). Cytochrome c acts as a cardiolipin oxygenase required for release of proapoptotic factors.[see comment]. Nat Chem Biol.

[22] Han X, Yang J, Cheng H, Yang K, Abendschein DR, Gross RW (2005). Shotgun lipidomics identifies cardiolipin depletion in diabetic myocardium linking altered substrate utilization with mitochondrial dysfunction. Biochemistry.

[23] Han X, Yang J, Yang K, Zhao Z, Abendschein DR, Gross RW (2007). Alterations in myocardial cardiolipin content and composition occur at the very earliest stages of diabetes: a shotgun lipodomics study. Biochemistry.

[24] Sparagna GC, Lesnefsky EJ (2009). Cardiolipin remodeling in the heart. J Cardiovasc Pharmacol.

[25] Menshikova EV, Ritov VB, Ferrell RE, Azuma K, Goodpaster BH, Kelley DE (2007). Characteristics of skeletal muscle mitochondrial biogenesis induced by moderate-intensity exercise and weight loss in obesity. J Appl Physiol.

[26] Lee HJ, Mayette J, Rapoport SI, Bazinet RP (2006). Selective remodeling of cardiolipin fatty acids in the aged rat heart. Lipids Health Dis.

[27] Orrenius S, Zhivotovsky B, Nicotera P (2003). Regulation of cell death: the calcium-apoptosis link. Nat Rev Mol Cell Biol.

[28] Paradies G, Petrosillo G, Pistolese M, Ruggiero FM (2002). Reactive oxygen species affect mitochondrial electron transport complex I activity through oxidative cardiolipin damage. Gene.

[29] Nomura K, Imai H, Koumura T, Kobayashi T, Nakagawa Y (2000). Mitochondrial phospholipid hydroperoxide glutathione peroxidase inhibits the release of cytochrome c from mitochondria by suppressing the peroxidation of cardiolipin in hypoglycaemia-induced apoptosis. Biochem J.

[30] Zhong Q, Gohil VM, Ma L, Greenberg ML (2004). Absence of cardiolipin results in temperature sensitivity, respiratory defects, and mitochondrial DNA instability independent of pet56. J Biol Chem.

[31] Ohtsuka T, Nishijima M, Suzuki K, Akamatsu Y (1993). Mitochondrial dysfunction of a cultured Chinese hamster ovary cell mutant deficient in cardiolipin. J Biol Chem.

[32] Schlame M, Brody S, Hostetler KY (1993). Mitochondrial cardiolipin in diverse eukaryotes. Comparison of biosynthetic reactions and molecular acyl species. Eur J Biochem.

[33] Schlame M, Horvath L, Vigh L (1990). Relationship between lipid saturation and lipid-protein interaction in liver mitochondria modified by catalytic hydrogenation with reference to cardiolipin molecular species. Biochem J.

[34] Yamaoka-Koseki S, Urade R, Kito M (1991). Cardiolipins from rats fed different dietary lipids affect bovine heart cytochrome c oxidase activity. J Nutr.

[35] Hostetler KY, Galesloot JM, Boer P, Van Den Bosch H (1975). Further studies on the formation of cardiolipin and phosphatidylglycerol in rat liver mitochondria. Effect of divalent cations and the fatty acid composition of CDP-diglyceride. Biochim Biophys Acta.

[36] Rustow B, Schlame M, Rabe H, Reichmann G, Kunze D (1989). Species pattern of phosphatidic acid, diacylglycerol, CDP-diacylglycerol and phosphatidylglycerol synthesized de novo in rat liver mitochondria. Biochim Biophys Acta.

[37] Schlame M, Rustow B, Kunze D, Rabe H, Reichmann G (1986). Phosphatidylglycerol of rat lung. Intracellular sites of formation de novo and acyl species pattern in mitochondria, microsomes and surfactant. Biochem J.

[38] Chen D, Zhang XY, Shi Y (2006). Identification and functional characterization of hCLS1, a human cardiolipin synthase localized in mitochondria. Biochem J.

[39] Houtkooper RH, Akbari H, van Lenthe H, Kulik W, Wanders RJA, Frentzen M (2006). Identification and characterization of human cardiolipin synthase. FEBS Lett.

[40] Lu B, Xu FY, Jiang YJ, Choy PC, Hatch GM, Grunfeld C (2006). Cloning and characterization of a cDNA encoding human cardiolipin synthase (hCLS1). J Lipid Res.

[41] Nie J, Hao X, Chen D, Han X, Chang Z, Shi Y (2009). A novel function of the human CLS1 in phosphatidylglycerol synthesis and remodeling. Biochim Biophys Acta.

[42] Taylor WA, Hatch GM (2003). Purification and characterization of monolysocardiolipin acyltransferase from pig liver mitochondria. J Biol Chem.

[43] Van Q, Liu J, Lu B, Feingold KR, Shi Y, Lee RM, Hatch GM (2007). Phospholipid scramblase-3 regulates cardiolipin de novo biosynthesis and its resynthesis in growing HeLa cells. Biochem J.

[44] Evans JL, Goldfine ID, Maddux BA, Grodsky GM (2002). Oxidative stress and stress-activated signaling pathways: a unifying hypothesis of type 2 diabetes. Endo Rev.

[45] Paradies G, Petrosillo G, Pistolese M, Di Venosa N, Federici A, Ruggiero FM (2004). Decrease in mitochondrial complex I activity in ischemic/reperfused rat heart: involvement of reactive oxygen species and CL. Cir Res.

[46] Pfeiffer K, Gohil V, Stuart RA, Hunte C, Brandt U, Greenberg ML (2003). Cardiolipin stabilizes respiratory chain supercomplexes. J Biol Chem.

[47] Paradies G, Petrosillo G, Pistolese M, Ruggiero FM (2000). The effect of reactive oxygen species generated from the mitochondrial electron transport chain on the cytochrome c oxidase activity and on the cardiolipin content in bovine heart submitochondrial particles. FEBS Lett.

[48] Barth PG, Valianpour F, Bowen VM, Lam J, Duran M, Vaz FM (2004). X-linked cardioskeletal myopathy and neutropenia (Barth syndrome): an update. Am J Med Genet Part A.

[49] Neuwald AF (1997). Barth syndrome may be due to an acyltransferase deficiency. Curr Biol.

[50] Barth PG, Scholte HR, Berden JA, Van der Klei-Van Moorsel J M, Luyt-Houwen IE, Van't Veer-Korthof E T (1983). An X-linked mitochondrial disease affecting cardiac muscle, skeletal muscle and neutrophil leucocytes. J Neurol Sci.

[51] Vreken P, Valianpour F, Nijtmans LG, Grivell LA, Plecko B, Wanders RJ (2000). Defective remodeling of cardiolipin and phosphatidylglycerol in Barth syndrome. Biochem Biophys Res Commun.

[52] Schlame M, Towbin JA, Heerdt PM, Jehle R, DiMauro S, Blanck TJ (2002). Deficiency of tetralinoleoyl-cardiolipin in Barth syndrome. Ann Neurol.

[53] Bissler JJ, Tsoras M, Goring HH, Hug P, Chuck G, Tombragel E (2002). Infantile dilated X-linked cardiomyopathy, G4.5 mutations, altered lipids, and ultrastructural malformations of mitochondria in heart, liver, and skeletal muscle. Lab Invest.

[54] Anderson EJ, Lustig ME, Boyle KE, Woodlief TL, Kane DA, Lin CT (2009). Mitochondrial H2O2 emission and cellular redox state link excess fat intake to insulin resistance in both rodents and humans. J Clin Invest.

[55] Bonnard C, Durand A, Peyrol S, Chanseaume E, Chauvin M-A, Morio B (2008). Mitochondrial dysfunction results from oxidative stress in the skeletal muscle of diet-induced insulin-resistant mice. J Clin Invest.

[56] Furukawa S, Fujita T, Shimabukuro M, Iwaki M, Yamada Y, Nakajima Y (2004). Increased oxidative stress in obesity and its impact on metabolic syndrome. J Clin Invest.

[57] Houstis N, Rosen ED, Lander ES (2006). Reactive oxygen species have a causal role in multiple forms of insulin resistance. Nature.

[58] Atabek ME, Vatansev H, Erkul I (2004). Oxidative stress in childhood obesity. J Pediatr Endocrinol Metab.

[59] Maxwell SR, Thomason H, Sandler D, Leguen C, Baxter MA, Thorpe GH (1997). Antioxidant status in patients with uncomplicated insulin-dependent and non-insulin-dependent diabetes mellitus. Eur J Clin Invest.

[60] Maddux BA, See W, Lawrence JC, Goldfine AL, Goldfine ID, Evans JL (2001). Protection against oxidative stress-induced insulin resistance in rat L6 muscle cells by mircomolar concentrations of alpha-lipoic acid. Diabetes.

[61] Tirosh A, Potashnik R, Bashan N, Rudich A (1999). Oxidative stress disrupts insulin-induced cellular redistribution of insulin receptor substrate-1 and phosphatidylinositol 3-kinase in 3T3-L1 adipocytes. A putative cellular mechanism for impaired protein kinase B activation and GLUT4 translocation. J Biol Chem.

[62] Ye G, Metreveli NS, Donthi RV, Xia S, Xu M, Carlson EC (2004). Catalase protects cardiomyocyte function in models of type 1 and type 2 diabetes. Diabetes.

[63] Watkins SM, Reifsnyder PR, Pan HJ, German JB, Leiter EH (2002). Lipid metabolome-wide effects of the PPARgamma agonist rosiglitazone. J Lipid Res.

[64] Hong MY, Chapkin RS, Barhoumi R, Burghardt RC, Turner ND, Henderson CE (2002). . Fish oil increases mitochondrial phospholipid unsaturation, upregulating reactive oxygen species and apoptosis in rat colonocytes. Carcinogenesis.

[65] Watkins SM, Carter LC, German JB (1998). Docosahexaenoic acid accumulates in cardiolipin and enhances HT-29 cell oxidant production. J Lipid Res.

[66] Pan HJ, Lin Y, Chen YE, Vance DE, Leiter EH (2006). Adverse hepatic and cardiac responses to rosiglitazone in a new mouse model of type 2 diabetes: relation to dysregulated phosphatidylcholine metabolism. Vascul Pharmacol.

[67] Yamaoka S, Urade R, Kito M (1988). Mitochondrial function in rats is affected by modification of membrane phospholipids with dietary sardine oil. J Nutr.

[68] Asayama K, Kato K (1990). Oxidative muscular injury and its relevance to hyperthyroidism. Free Radical Biol Med.

[69] Nishiki K, Erecinska M, Wilson DF, Cooper S (1978). Evaluation of oxidative phosphorylation in hearts from euthyroid, hypothyroid, and hyperthyroid rats. Am J Physiol.

[70] Venditti P, De Rosa R, Cigliano L, Agnisola C, Di Meo S (2004). Role of nitric oxide in the functional response to ischemia-reperfusion of heart mitochondria from hyperthyroid rats. Cell Mol Life Sci.

[71] Hatch GM (2004). Cell biology of cardiac mitochondrial phospholipids. Biochem Cell Biol.

[72] Gredilla R, Lopez Torres M, Portero-Otin M, Pamplona R, Barja G (2001). Influence of hyper- and hypothyroidism on lipid peroxidation, unsaturation of phospholipids, glutathione system and oxidative damage to nuclear and mitochondrial DNA in mice skeletal muscle. Mol Cell Biochem.

[73] Cao SG, Cheng P, Angel A, Hatch GM (1995). Thyroxine stimulates phosphatidylglycerolphosphate synthase activity in rat heart mitochondria. Biochim Biophys Acta.

[74] Hostetler KY (1991). Effect of thyroxine on the activity of mitochondrial cardiolipin synthase in rat liver. Biochim Biophys Acta.

[75] Mutter T, Dolinsky VW, Ma BJ, Taylor WA, Hatch GM (2000). Thyroxine regulation of monolysocardiolipin acyltransferase activity in rat heart. Biochem J.

[76] Taylor WA, Xu FY, Ma BJ, Mutter TC, Dolinsky VW, Hatch GM (2002). Expression of monolysoCL acyltransferase activity is regulated in concert with the level of cardiolipin and cardiolipin biosynthesis in the mammalian heart. BMC Biochem.

[77] Venditti P, Di Meo S (2006). Thyroid hormone-induced oxidative stress. Cell Mol Life Sci.

[78] Cao J, Shen W, Chang Z, Shi Y (2009). ALCAT1 is a polyglycerophospholipid acyltransferase potently regulated by adenine nucleotide and thyroid status. Am J Physiol Endocrinol Metab.

[79] Turrens JF (2003). Mitochondrial formation of reactive oxygen species. J Physiol.

[80] Papa S, Skulachev VP (1997). Reactive oxygen species, mitochondria, apoptosis and aging. Mol Cell Biochem.

[81] Paradies G, Petrosillo G, Pistolese M, Ruggiero FM (2001). Reactive oxygen species generated by the mitochondrial respiratory chain affect the complex III activity via cardiolipin peroxidation in beef-heart submitochondrial particles. Mitochondrion.

[82] Petrosillo G, Ruggiero FM, Di Venosa N, Paradies G (2003). Decreased complex III activity in mitochondria isolated from rat heart subjected to ischemia and reperfusion: role of reactive oxygen species and cardiolipin. FASEB J.

[83] Pierangeli SS, Harris EN (2003). Probing antiphospholipid-mediated thrombosis: the interplay between anticardiolipin antibodies and endothelial cells. Lupus.

[84] Sparagna GC, Chicco AJ, Murphy RC, Bristow MR, Johnson CA, Rees ML (2007). Loss of cardiac tetralinoleoyl cardiopilin in human and experimental heart failure. J Lipid Res.

[85] Shigenaga MK, Hagen TM, Ames BN (1994). Oxidative damage and mitochondrial decay in aging. Proc Natl Acad Sci USA.

[86] Wallace DC (2005). A mitochondrial paradigm of metabolic and degenerative diseases, aging, and cancer: a dawn for evolutionary medicine. Annu Rev Genet.

[87] Pamplona R, Portero-Otin M, Riba D, Ruiz C, Prat J, Bellmunt MJ (1998). Mitochondrial membrane peroxidizability index is inversely related to maximum life span in mammals. J Lipid Res.

[88] Hulbert AJ (2008). The links between membrane composition, metabolic rate and lifespan. Comp Biochem Physiol A Mol Integr Physiol.

[89] Wright AF, Jacobson SG, Cideciyan AV, Roman AJ, Shu X, Vlachantoni D (2004). Lifespan and mitochondrial control of neurodegeneration. Nat Genet.

[90] Paradies G, Ruggiero FM (1991). Effect of aging on the activity of the phosphate carrier and on the lipid composition in rat liver mitochondria. Arch Biochem Biophy.

[91] Paradies G, Ruggiero FM, Gadaleta MN, Quagliariello E (1992). The effect of aging and acetyl-L-carnitine on the activity of the phosphate carrier and on the phospholipid composition in rat heart mitochondria. Bioch Biophy Acta.

[92] Paradies G, Ruggiero FM, Petrosillo G, Gadaleta MN, Quagliariello E (1994). Effect of aging and acetyl-L-carnitine on the activity of cytochrome oxidase and adenine nucleotide translocase in rat heart mitochondria. FEBS Lett.

[93] Paradies G, Ruggiero FM, Petrosillo G, Quagliariello E (1997). Age-dependent decline in the cytochrome c oxidase activity in rat heart mitochondria: role of cardiolipin. FEBS Lett.

[94] Paradies G, Petrosillo G, Gadaleta MN, Ruggiero FM (1999). The effect of aging and acetyl-L-carnitine on the pyruvate transport and oxidation in rat heart mitochondria. FEBS Lett.

[95] Pope S, Land JM, Heales SJ (2008). Oxidative stress and mitochondrial dysfunction in neurodegeneration; Cardiolipin a critical target?. Biochim Biophys Acta.

[96] Ruggiero FM, Cafagna F, Petruzzella V, Gadaleta MN, Quagliariello E (1992). Lipid composition in synaptic and nonsynaptic mitochondria from rat brains and effect of aging. J Neurochem.

[97] Sen T, Sen N, Tripathi G, Chatterjee U, Chakrabarti S (2006). Lipid peroxidation associated cardiolipin loss and membrane depolarization in rat brain mitochondria. Neurochem Int.

[98] Kiebish MA, Han X, Cheng H, Chuang JH, Seyfried TN (2008). Cardiolipin and electron transport chain abnormalities in mouse brain tumor mitochondria: lipidomic evidence supporting the Warburg theory of cancer. J Lipid Res.

[99] Ellis CE, Murphy EJ, Mitchell DC, Golovko MY, Scaglia F, Barcelo-Coblijn GC (2005). Mitochondrial lipid abnormality and electron transport chain impairment in mice lacking alpha-synuclein. Mol Cell Biol.

[100] Bayir H, Tyurin VA, Tyurina YY, Viner R, Ritov VB, Amoscato AA (2007). Selective early cardiolipin peroxidation after traumatic brain injury: an oxidative lipidomics analysis. Ann Neurol.

